# Lichens – growing greenhouses *en miniature*

**DOI:** 10.15698/mic2021.03.743

**Published:** 2021-03-01

**Authors:** Martin Grube

**Affiliations:** 1 Institute of Biology, University of Graz, Graz, Austria.

**Keywords:** fungi, microbiome, symbiosis, complexity, poikilohydry

**Figure 1:**
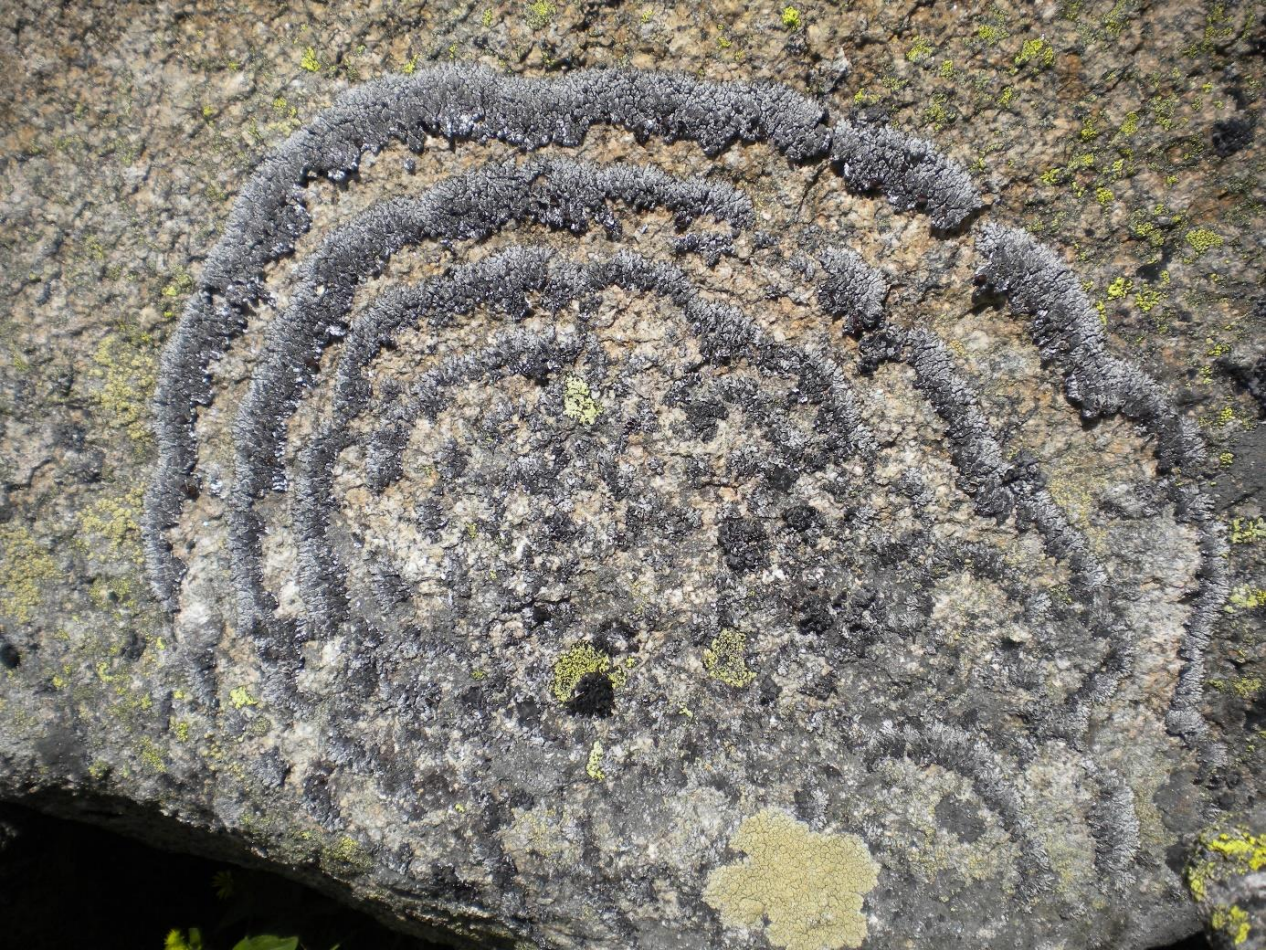
FIGURE 1: *Brodoa atrofusca*, alpine rock inhabiting lichen frequently growing in wave-like concentric fashion. Thallus diameter c. 60 cm (Hohe Tauern, Austria. Foto: Martin Grube).

Beards hanging from trees and colorful patches encrusting rocks are silent success stories of lichens, the fascinating life styles fungi can form with algae (**Fig. 1**). Lichens were show-cases to introduce the concept of symbiosis (as ‘Symbiotismus' [1]). The self-support of symbiotic life styles is recognized as gear-shift of evolution and applied to a vast number of examples where continued interactions between species lead to metabolic or phenotypic novelty. Lichen symbioses are still outstanding for the structural longevity and occurrence in environments, some which are unsuitable for most other organisms. Lichens often form major components Arctic tundra, boreal forest floors, but also on lava fields, rock surfaces along coasts or in extremely high altitudes. The perseverance of lichens in such hostile places appears to be in striking contrast to observed ecological specialization and their lack in urban and trafficated places. The symbiosis is indeed very sensitive during physiologically active state but the puzzle of extremotolerance is solved when we consider poikilohydry: because lichens hardly possess structural or functional mechanisms to maintain and/or regulate water content, desiccation rapidly causes shut down of metabolism. Yet, in contrast to many other life forms, lichens cope extremely well with recurrent changes of water availability.

Lichens have an outstanding ability to revitalize from dry stages. Lichens can endure extreme desiccation to water contents (below 0.1 g H_2_O g–1 dry weight (DW)), which causes ‘vitrification', the transition of their cytoplasm to a ‘glassy' state and cease of metabolism. To find out what reactions may occur at different levels of desiccation in lichens, Candotto Carniel *et al.* [2] used dynamic mechanical thermal analysis as for assessment of molecular mobility, while de- and re-epoxidation of the xanthophyll cycle pigments served as a proxy to assess enzyme activity. At 20°C vitrification occurred between 0.12–0.08 g H_2_O g–1 DW and enzymes were active in a ‘rubbery' state (0.17 g H_2_O g–1 DW) but not in a glassy state (0.03 g H_2_O g–1 DW). Therefore, desiccated tissues may appear to be ‘dry' in the conventional sense, but subtle differences in water content will have substantial consequences on the types of (bio)chemical reactions that can occur, with downstream effects on longevity in the desiccated state.

Lichen thalli must be flexible to retain shape integrity under poikilohydric conditions, which involve shrinking and swelling of the symbiotic structures. The photosynthetic partners in the majority of lichens, algae or cyanobacteria, are typically sheltered beneath coherent peripheral layers formed by fungal cells, which are tightly glued together in a common extracellular matrix by their gelatinizing outer cell walls. Spribille *et al.* [3] compiled current knowledge about the composition of involved polysaccharides and emphasized the important role of acidic polysaccharides in holding lichens together. The potential effects of desiccation and rewetting (D/W) cycles for regulation of fungal polysaccharide composition still needs to be established. For the algal partner of lichens, González-Hourcade *et al.* [4] already discovered that exposure to D/W cycles strongly altered the size distribution of certain polysaccharides. The authors concluded that biochemical remodeling of the cell wall could increase flexibility, allowing regulated shrinkage and expansion of algal symbionts. Lack of characteristic environmental triggers, including D/W cycles, might also explain why native thallus structures lichens are hardly re-synthesized in Petri dish cultures.

Several attempts have recently been undertaken to achieve a better understanding of the genomic “hardwiring” for the lichen symbiosis. For example, Armaleo *et al.* [5] conducted a first parallel genomic analysis of the mycobiont *Cladonia grayi* and of its green algal photobiont *Asterochloris glomerata.* Gene family expansions were present in both symbionts (such as, signal transduction components, ankyrin domain proteins and transcription factors involved in chromatin remodeling and stress responses), as well as expanded fungal protein families (such as heterokaryon incompatibility proteins, polyketide synthases, and a unique set of G-protein α subunit paralogs) and expanded algal protein families (carbohydrate active enzymes and a specific subclass of cytoplasmic carbonic anhydrases). Horizontal gene transfer from prokaryotes played a likely role for acquisition of novel archaeal ATPases and Desiccation-Related Proteins by the algae. According to these results lichens evolved by accretion of many scattered regulatory and structural changes, which agrees with an independent origin of lichenized fungal lineages in the fungal kingdom. Kono *et al.* [6] succeeded in resynthesizing tiny symbiotic stages of the beard lichen *Usnea hakonensis* for transcriptomic analyses. By comparing resynthesized and natural thalli (symbiotic states) with that of isolated cultures (non-symbiotic state), they found evidence for various processes involved in symbiotic establishment, including cell wall remodeling, production of hydrophobins (which seal an apoplastic continuum of interacting cells of fungi and algae) and symbiosis-specific nutrient flow (including polyol transporters). Future transcriptomic approaches need to consider a side by side of life and death in lichens. Behind the growing thallus edges, even neighbouring cells can vary in vitality and fungal remnants are often part of protective surface layers or provide a scaffold for a fraction of living cells. In some lichens, these layers contain inflated algal cell walls as well, while in others, the remnant algal walls stay put and supposedly support the architectural integrity of thalli. New results, such as the detection of caspase-like activity as a marker of programmed cell death in lichens [7], opens many new questions about the organization of vitality in long persisting thallus structures.

**Figure 2:**
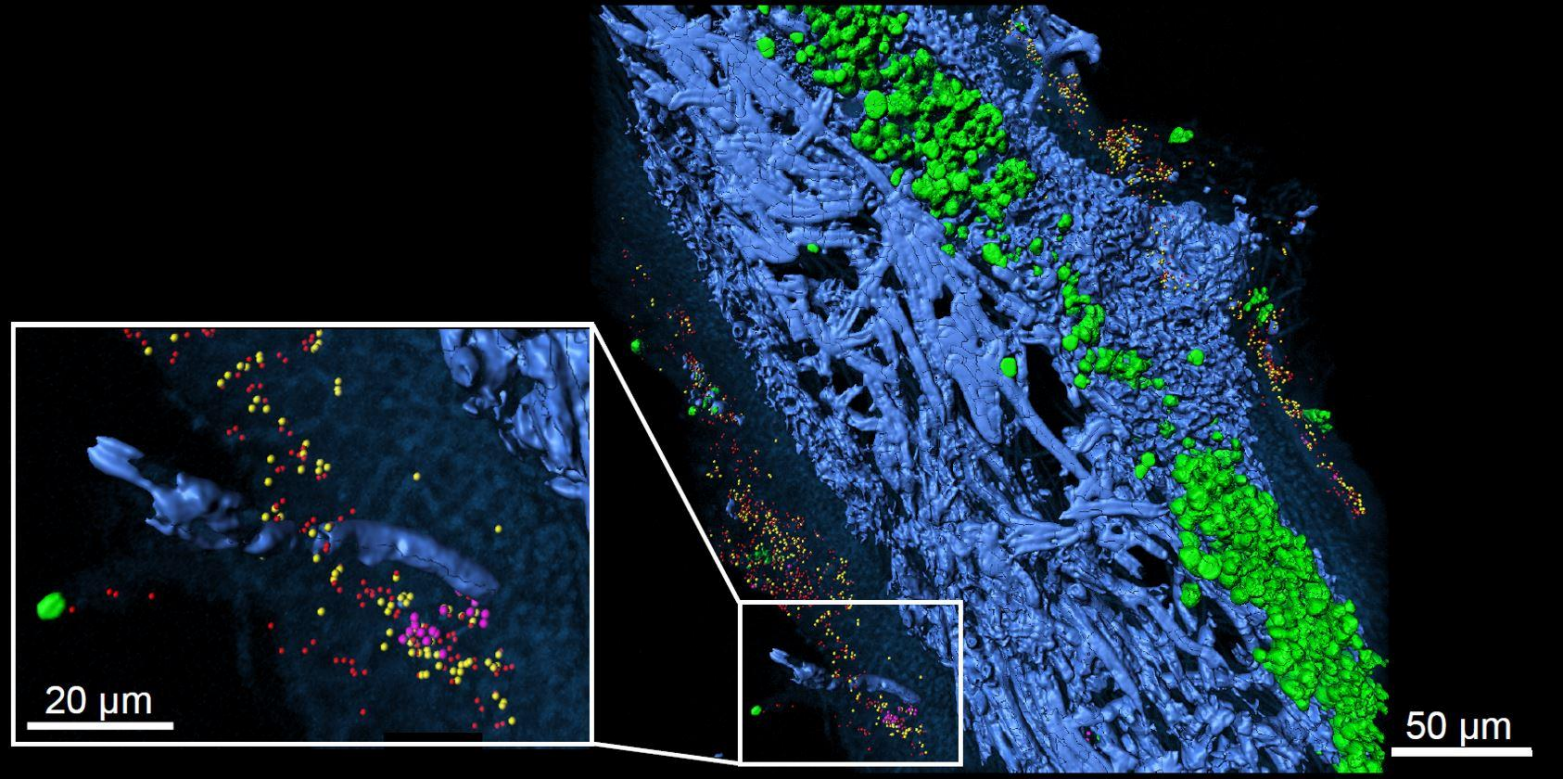
FIGURE 2: Lichen thallus structure of lung lichen *Lobaria pulmonaria* in cross-section. 3D reconstruction of FISH image stacks. Eubacteria (red) and Alphaproteobacteria (yellow) were found widespread on both, the upper and the lower cortex, while Betaproteobacteria (pink) were less abundant and locally contained. Fungal hyphae (blue) and algae located under the upper cortex (green) are visualized by their autofluorescence (from [10] with permission of Springer Nature).

Over the past decade it has also become increasingly clear that the living-together of fungi and algae is more complex than previously thought. Algal partners are not uniform and may switch with climatic parameters, while the lichen phenotypes also host associated microbiota (**Fig. 2**), which is considered in a recently emended definition of the lichen symbiosis [8]. While new information suggests a widespread occurrence of yeasts in lichens, their role is still unclear [9]. With respect to bacteria, Grube *et al.* [10] published a comprehensive overview about diversity and the potential functional roles of the bacterial microbiome using metagenomic and metaproteomic approaches. In addition, metatranscriptomic analyses revealed transcriptional responses of bacterial microbiota in relation to water status, indicating a switch to lipid-based nutrition of associated bacteria in dry lichens [11]. Despite these progresses, host-specific factors controlling bacterial composition are not well known. The residing bacterial fraction on the surfaces underlies the same stressful conditions as the host lichen, but also to regular oxidative bursts of the lichen during rewetting [12]. In addition to potential biotrophic contributions of bacteria, it needs to be tested whether decaying bacterial fractions could end up resorbed for nutrients by the host. Initial colonization depends on habitat-specific condition such as adjacent substrates, but factors including surface hydrophobicity and or chemical composition contribute to the specificity of microbiota on lichens [13].

The varied colors of lichens are the result of accumulations of crystallized metabolites, which deposited on the outside of the fungal cells as light filters or herbivore deterrents. Thousands of compounds are known but only few are better characterized for antibiotic effects and other bioactive potentials [14]. Research is now directed towards characterization of potential candidate genes from enriched families of biosynthetic genes in lichen fungal genomes. For example, usnic acid is a widespread polyketide produced by lichens with antibacterial, antiviral, and antitumor bioactive properties. Transcriptome analysis of *Nephromopsis pallescens* revealed one of nine found type I PKS, *Nppks7,* as potentially involved in usnic acid biosynthesis [15]. Nppks7 is a non-reducing polyketide synthase with a MeT domain that also possesses beta-ketoacyl-ACP synthase, acyl transferase, product template, acyl carrier protein, C-methyltransferase, and Claisen cyclase domains. However, tests using knock out mutants fail due to lack of efficient protocols for lichens and biosynthesis of specific lichen compound via heterologous expressions has not yet successfully been achieved due to problems of translation [16]. The dozens of biosynthetic genes present in each of the lichen fungal genomes [15], as well as those of their microbial associates, call for targeted imaging metabolomics to reveal spatial distribution of actually produced metabolites [17].

Cultivation of genuine lichens has remained difficult, but enhanced productivity of fungal-algal associations has been demonstrated also for co-cultures of faster-growing partners in the laboratory. Such studies also direct to hitherto unknown beneficial effects in the lichen symbiosis. As Krespach *et al.* [18] discovered, cooperation with fungi could help algae to survive in environments with potentially lethal other microorganisms. The co-cultured mycelium of *Aspergillus nidulans* shields the alga *Chlamydomonas reinhardtii* from toxic azalomycin F produced by the bacterium *Streptomyces iranensis*. Whether similar effects could be part of the ecological success of lichen phenotypes in their natural environments still remains to be studied. Considering the high potential of compound production in lichens, science would be ready for further surprising discoveries. While the pocket-sized microbial ecosystems of lichens are still a challenge for research, new technological approaches provide bridgeheads for culture-independent functional studies.
